# Coping Strategies, Self-Efficacy and Their Relationship with Anxiety and Depression in Early Childhood Care Professionals

**DOI:** 10.3390/healthcare14050609

**Published:** 2026-02-27

**Authors:** María Guillot-Valdés, Sofía Gómez-Herrera, María Auxiliadora Robles-Bello, Nieves Valencia-Naranjo, María Eva Martín-Puga, David Sánchez-Teruel

**Affiliations:** 1 Faculty of Psychology, University of Jaen, 23071 Jaen, Spain; mguillot@ujaen.es (M.G.-V.); sgh00013@red.ujaen.es (S.G.-H.); nnaranjo@ujaen.es (N.V.-N.); emartin@ujaen.es (M.E.M.-P.); 2 Faculty of Psychology, University of Granada, 18012 Granada, Spain; dsteruel@ugr.es

**Keywords:** early intervention, mental health, coping, self-efficacy, psychological distress

## Abstract

**Background/Objectives**: The mental health of Early Childhood Care professionals is of great importance to ensuring the quality of intervention and the well-being of families. The aim of this study was to analyze the relationship between coping strategies, perceived self-efficacy and levels of depression, anxiety and stress in Early Childhood Care professionals. **Methods**: A study was conducted with a sample of 125 professionals (87% women; M = 33.40, SD = 9.70). Participants completed the Coping Strategies Inventory, the General Self-Efficacy Scale, and the DASS-21. The sample was predominantly female, which should be considered when interpreting the findings. **Results:** Cognitive restructuring, positive restructuring, and social support were negatively associated with depression, anxiety, and stress, while social withdrawal was positively related to all these variables. Self-efficacy showed high negative correlations with psychological distress and was a strong protective predictor. Emotional expression showed a positive association with depression. **Conclusions**: Self-efficacy and adaptive coping strategies act as protective factors against psychological distress, while social withdrawal is a significant risk. These findings highlight the need to implement training and prevention programs primarily aimed at enhancing perceived self-efficacy, with adaptive coping strategies acting as behavioral mechanisms through which this protective factor is strengthened and maladaptive responses are reduced.

## 1. Introduction

Mental health is an essential component of individual well-being and professional performance. According to the World Health Organization-WHO [1], mental health is a state of well-being that enables people to cope with daily stress, develop their abilities, work and learn effectively, and contribute to their community; it is an essential human right. Although mental disorder affects approximately one in eight people worldwide, most commonly depression or anxiety, less than 2% of state budgets are allocated to mental health, according to estimates from the Pan American Health Organization [2]. This lack of investment critically limits specialised care, especially in low- and middle-income countries, where the ratio of trained professionals is extremely low; in some cases, a single psychiatrist cares for more than 200,000 people, highlighting the urgent need to increase specialised mental health human resources globally [3].

This challenge is particularly relevant in the healthcare sector, given that the psychological well-being of its professionals has a direct impact on the quality of care provided. Recent evidence has shown that the emotional distress of healthcare workers reduces the quality of care, impairs the user experience and increases the risk of errors, thus compromising patient safety and well-being [4]. The COVID-19 pandemic has exacerbated this situation: globally, 23% of healthcare professionals reported symptoms of depression and anxiety, and 39% suffered from insomnia, highlighting the vulnerability of this population to the stress associated with this type of work [2,5]. Psychological distress increases in critical units, such as emergency rooms or intensive care units, where professionals face additional risks, as well as in the field of mental health, where stressors such as exposure to negative emotions, suicidal thoughts, or extensive bureaucratic procedures accumulate [6,7,8]. In addition to anxiety and depression, stress was included because these constructs are considered part of a broader emotional distress continuum in occupational mental health models. In healthcare professionals, chronic work-related stress frequently acts as a precursor and maintaining factor for anxiety and depressive symptoms [9,10]. Therefore, the DASS-21 stress subscale was incorporated to capture the activation component of psychological distress and to better understand the relationship between coping, self-efficacy and overall emotional adjustment.

In this context, Early Childhood Care Centers (ECC) take on special relevance in Spain, involving multidisciplinary teams who work with children aged 0–6 years with developmental disorders or at risk of developing them, and with their families. These professionals face significant emotional demands, including the communication of diagnostic information, which can in turn cause emotional distress for the families [11,12,13]. The support provided by these professionals is a determining factor in the early development outcomes of children [14]. Therefore, it is crucial to investigate the mental health of ECC professionals and the protective factors that can mitigate the impact of these demands, given that there is a significant empirical gap in this sector [15]. Notably, research conducted by Gómez-Herrera et al. [16] indicates that there are currently no scientific studies specifically addressing the mental health and resilience of Early Childhood Care professionals. Consequently, the following discussion draws on research conducted with healthcare professionals, as this represents the closest available evidence to inform understanding in the ECC context.

Several studies have examined the role of coping strategies in protecting healthcare professionals from psychological distress. Traditionally, Lazarus and Folkman [17,18] defined coping strategies as cognitive, emotional, and behavioral efforts that people use to manage demands perceived as overwhelming. Classic evidence shows that strategies focused on internal management and emotional regulation, together with resilience, are more adaptive than avoidance strategies and are associated with lower levels of anxiety, stress, and depression [19,20]. Recent studies confirm and expand on these findings: avoidance and stress perception strategies are linked to a higher risk of emotional distress, while job satisfaction acts as a protective factor [21]. It has also been concluded that denial, substance use and cessation of activities are common strategies among healthcare professionals, while acceptance is rarely used. This type of behaviour can predict the long-term deterioration of the mental state of these professionals [22]. On the contrary, mechanisms focused on emotional regulation are related to anxiety and depression, while problem-oriented strategies are more adaptive [23]. Likewise, studies affirm that there are variations in coping and perceived stress among healthcare professionals depending on the type of workplace—hospitals versus health centers [24].

Complementarily, self-efficacy has been shown to play a key role in protecting against work-related distress and emotional exhaustion. According to Bandura [25,26], self-efficacy is defined as the belief in one’s own ability to organize and execute actions in challenging situations. Classic studies in the healthcare field show that positive self-efficacy is associated with greater subjective well-being and job satisfaction, while low self-efficacy correlates with anxiety, depression, and burnout [27,28]. Recent findings reinforce these results: self-efficacy has a direct effect on mental health in professionals working in patient care and is capable of reducing perceived stress and emotional exhaustion even in high-pressure contexts, such as the COVID-19 pandemic [29,30,31]. In addition, patient-centred self-efficacy improves satisfaction and adherence to treatment, enhancing the effectiveness of chronic disease management [32]. Likewise, it has been observed that nursing staff self-efficacy correlates negatively with emotional exhaustion and can predict burnout along with high levels of anxiety, underscoring the importance of interventions aimed at strengthening self-efficacy [33].

In summary, the mental health of healthcare professionals, particularly those working in ECC, is critical to ensuring adequate child development and family well-being. Despite abundant evidence in other healthcare settings, the literature on this group is limited. Given this gap, it is necessary to explore the role of protective factors in mitigating psychological distress, as effective public healthcare is only possible if it has a competent, motivated and resilient professional workforce that ensures the quality of care and the sustainability of the system [34]. Therefore, the present study aims to determine whether coping strategies and self-efficacy are related to psychological distress in early childhood healthcare professionals. Finally, it determines which of these factors are most important in terms of predictive capacity. It is expected that participants who scored low on coping strategies and self-efficacy will score high on depression, anxiety and stress.

## 2. Materials and Methods

### 2.1. Participants

The sample consists of 125 professionals (87% women), aged between 23 and 63 years, with a mean age of 33.4 years (SD = 9.70). Sampling was intentional. Table 1 presents a summary of other sociodemographic data. All participants signed an informed consent form and were informed about the characteristics of this study.

### 2.2. Evaluation Measures

The ad-hoc questionnaire measured the variables of gender, age, place of origin, marital status, profession, general education and place of work.

Coping Strategies Inventory, Spanish adaptation by Cano-García [35]. It consists of one open-ended question and 40 items. These are answered on a 5-point Likert scale (0 = not at all; 4 = completely). They are distributed across eight primary factors: (a) problem solving, (b) cognitive restructuring, (c) social support, (d) emotional expression, (e) problem avoidance, (f) wishful thinking, (g) social withdrawal, (h) self-criticism. Grouping the primary factors gives rise to the secondary strategies of: (a) adequate problem-focused coping (problem solving and cognitive restructuring), (b) adequate emotion-focused coping (social support and emotional expression), (c) inadequate problem-focused coping (problem avoidance and wishful thinking), (d) inadequate emotion-focused coping (social withdrawal and self-criticism). Although the instrument includes an initial open-ended question describing a stressful situation, responses to this item were not included in the analyses. Only quantitative Likert-scale items were used for statistical analysis in order to maintain methodological consistency across measures. The internal consistency indices of the Spanish adaptation ranged from 0.72 to 0.94.

General Self-efficacy Scale [36]. It consists of 10 items with a 4-point Likert scale. It assesses the stable feeling of personal competence to adequately handle different types of stressful situations. An overall score between 10 and 40 is obtained. In the Spanish adaptation by Suárez [37], internal consistency of (α) 0.87 was found. It has good convergent and discriminant validity and high test–retest reliability.

Depression Anxiety Stress Scale (DASS-21) [38]. It is used to assess emotional states of depression, anxiety, and stress. It consists of 21 items divided into three subscales (Depression, Anxiety, and Stress), each of which is composed of seven items, which are rated on a four-point Likert scale (from 0 = “does not apply to me at all” to 3 = “applies to me most of the time”). In its original study, this scale obtained an alpha (α) of 0.94 for Depression, 0.87 for Anxiety, and 0.91 for Stress.

### 2.3. Design and Procedure

This is a quantitative, descriptive-correlational, cross-sectional, non-experimental, ex post facto study. First, the necessary documentation to guarantee data confidentiality was sent to the Ethics Committee of the University of the corresponding author (reference JUN.23/3 PRY). Data collection began when the project was obtained at the end of 2025. An online questionnaire was created using Google Forms. Data collection continued during the study period. Participants were recruited through Early Childhood Care centers and professional networks. The research team contacted center coordinators and professional associations by email and provided information about the study objectives and voluntary participation. The survey link was distributed to professionals through institutional mailing lists and internal communication channels, and participation was anonymous and unpaid. Periodic reminders were sent to increase response rates. The informed consent form was attached to it as a prerequisite and mandatory requirement for taking the tests. Finally, the results were coded for subsequent analysis. Statistical analyses were performed using parametric procedures. Prior to conducting the regression analyses, multicollinearity among coping strategy variables was examined using Variance Inflation Factor (VIF) and tolerance statistics. All predictors showed acceptable values (VIF < 5; tolerance > 0.20), indicating that multicollinearity did not affect the interpretation of the regression coefficients. Statistical analysis was performed using the SPSS statistical package version 22.0.

## 3. Results

The assumption of normality for the main variables was calculated using the Kolmogorov–Smirnov test. Although some of them did not follow a normal distribution, given the sample size (nN = 125), parametric analyses were continued and Pearson correlations were used. Next, descriptive statistics were calculated for all subscales of the DASS-21, those of Coping Strategies and Self-Efficacy (Table 2).

As can be seen in Table 3, Pearson’s correlations between variables indicate that cognitive restructuring, social support, and positive restructuring correlate negatively and significantly with the three indicators of psychological distress, so that greater use of these strategies is associated with lower levels of depression, anxiety, and stress. On the other hand, social withdrawal shows moderate positive correlations with depression (r = 0.54, *p* < 0.01), anxiety (r = 0.55, *p* < 0.01) and stress (r = 0.42, *p* < 0.01), indicating that this strategy is associated with higher levels of distress.

For its part, self-efficacy shows significant negative correlations with all variables, being particularly high with depression (r = −0.59, *p* < 0.01), which supports its preventive role against psychological symptoms. Self-criticism and Paralysis by despair are also positively related to depression and anxiety, although with lower effect sizes. In contrast, emotional expression and avoidance show no significant correlations with virtually any of the variables.

Subsequently, a multiple linear regression analysis was performed to assess the extent to which self-efficacy and coping strategies predict levels of depression, anxiety, and stress (Table 4).

For the depression variable, the model was significant, F (9, 115) = 18.88, *p* < 0.001, and explained 59.6% of the variance in depression (R^2^ = 0.60, adjusted R^2^ = 0.57). The variables social withdrawal (β = 0.52, *p* < 0.001), total self-efficacy (β = −0.45, *p* < 0.001), and positive restructuring (β = −0.42, *p* < 0.001) were the most powerful predictors. Social support (β = −0.19, *p* = 0.02), emotional expression (β = 0.28, *p* < 0.001), and cognitive restructuring (β = −0.19, *p* = 0.04) were also significant. Other variables such as self-criticism and Paralysis by despair did not reach statistical significance.

Although emotional expression did not show a significant bivariate correlation with depression, it emerged as a significant predictor in the regression model. This pattern suggests a suppression effect. Therefore, its effect should be interpreted as conditional rather than direct.

A multiple regression analysis was also performed for the DASS-21 anxiety subscale. The model was significant, F (9, 115) = 12.80, *p* < 0.001, and explained 50% of the variance in anxiety (R^2^ = 0.50, adjusted R^2^ = 0.46). The variables positive restructuring (B = −0.19, *p* = 0.03) and social withdrawal (B = 0.51, *p* < 0.001) had significant associations with anxiety. Self-efficacy showed a trend, although not statistically significant (*p* = 0.06), as did problem-focused coping (PFC).

Finally, a multiple regression was performed for the Stress subscale. The model was significant, F (9, 115) = 11.01, *p* < 0.001, explaining 46.4% of the variance in stress levels (R^2^ = 0.46, adjusted R^2^ = 0.42). In this case, the variables that obtained the most significant associations with stress were Social Withdrawal (B = 0.37, *p* < 0.001) and Cognitive Restructuring (B = −0.34, *p* = 0.001). Self-efficacy was also significant (B = −0.29, *p* = 0.01). The rest of the variables did not obtain significant associations.

The regression models showed adequate explanatory power, with adjusted R^2^ values of 0.57 for depression, 0.46 for anxiety, and 0.42 for stress.

## 4. Discussion

The main objective of this study was to analyze the relationship between different coping strategies, perceived self-efficacy and levels of depression, anxiety and stress in early childhood care professionals. The aim was to see which coping styles act as protective or risk factors for psychological distress, as well as to determine the possible modulating role of self-efficacy.

Firstly, cognitive restructuring strategies, social support and positive restructuring have been shown to be associated with lower levels of depression, anxiety and stress. This is consistent with studies showing that cognitive restructuring helps to cushion the impact of stressful situations, such as the COVID-19 pandemic, and improve emotional recovery [39,40]. Similarly, social support has also been associated with higher levels of psychological well-being in special education professionals, complemented by the use of cognitive restructuring [41]. Cognitive restructuring has been linked to greater resilience and lower vulnerability to stress and depression in emotionally demanding contexts [40,42].

Self-efficacy has been found to be a protective factor against depression, anxiety, and stress. This result coincides with other studies, which have shown that self-efficacy acts as a mediator and buffer against the impact of stressors on mental health in areas such as social education, early childhood education and among parents of children with ASD [43,44,45,46,47]. This highlights the importance of promoting self-efficacy in these contexts in order to cope with difficult situations and increase the effectiveness of coping strategies.

A particularly interesting finding is that emotional expression showed a positive association with depression in this study, although it has usually been considered an adaptive strategy [48,49,50]. This finding could be due to the fact that, in emotionally demanding work contexts, the expression of negative emotions could increase rather than reduce distress in the absence of effective regulation strategies [51,52]. Thus, it would be important to distinguish between regulated and constructive emotional expression and excessive or repetitive expression that does not involve additional coping resources. This could mean that in certain contexts, such as early childhood healthcare, expressing emotions without using re-evaluation techniques or social support is not adaptive. The apparently contradictory finding—non-significant correlation but significant regression coefficient—may reflect a statistical suppression effect. Emotional expression shares variance with both adaptive and maladaptive strategies; when overlapping variance is removed, its unique contribution becomes observable. This suggests that emotional expression is not inherently adaptive or maladaptive, but depends on the regulatory context in which it occurs.

In summary, the results highlight the importance of promoting training and prevention programs that enhance self-efficacy and promote adaptive coping strategies among professionals working in the field of early childhood care. Such programs could include training in cognitive restructuring skills, strengthening support networks and problem-solving techniques, as well as interventions aimed at reducing the use of avoidance strategies [53]. To enhance practical applicability, the intervention model can be structured into a brief institutional program integrated into routine professional training [54,55]. The program would consist of four modules delivered over 6–8 weeks: (1) psychoeducation on emotional demands in early childhood care [11,14,53]; (2) cognitive restructuring and emotional regulation training using real clinical scenarios [51,52]; (3) structured problem-solving workshops focused on communication with families [11,14]; (4) supervised peer reflection groups aimed at preventing social withdrawal and reinforcing perceived self-efficacy [27,33].

The program could be implemented in small interdisciplinary groups (6–10 professionals) in either face-to-face or online format, requiring approximately 2 h biweekly [54,55,56]. Additionally, differentiated emphasis may be applied depending on professional role: therapists (emotion regulation), psychologists (family communication), and coordinators (team support and early detection of withdrawal behaviors) [27,33].

Interprofessional interventions in hospitals, such as the Adaptive Care program, have been shown to increase self-efficacy, confidence and the variety of coping strategies among nursing staff caring for children with special needs, improving the quality of care and the working environment [57]. Therefore, a practical course of action would be to adapt similar programs to the context of Early Childhood Care, combining psychosocial support with more technical training.

However, this study is not without limitations. First, the cross-sectional design prevents the establishment of causal relationships between variables. Secondly, the exclusive use of self-reports could cause biases such as social desirability or subjective perception of one’s own coping skills. In addition, the strong gender imbalance in the sample should be considered when interpreting the findings. Previous research indicates gender differences in coping styles, perceived self-efficacy and vulnerability to anxiety and depressive symptoms. Therefore, the predictive relationships observed in this study may not operate in the same way in predominantly male professional populations, limiting the generalizability of the results. In future studies, it would be advisable to expand the sample to include greater gender diversity and a wider range of educational and healthcare profiles. It should also include mixed methodologies, such as qualitative interviews or observational methods, in order to study how professionals apply their coping techniques in real life more comprehensively.

Another limitation concerns the geographical concentration of the sample. Although participants came from several Spanish regions, more than half were recruited from a single province, and all professionals worked within the Spanish Early Childhood Care system. Differences in organizational structure, resource availability, and intervention models (e.g., public vs. private services) may influence coping dynamics and psychological distress. Therefore, the findings should be generalized with caution to other healthcare systems or cultural contexts. Future studies should include multicenter and cross-national samples to examine whether the predictive role of coping strategies and self-efficacy remains stable across organizational models and cultural contexts. Comparative analyses between public and private services would be particularly relevant to determine the influence of institutional factors on professional well-being.

Future research could also benefit from a longitudinal design that allows for the analysis of the evolution of coping strategies and self-efficacy in relation to psychological distress, as well as the evaluation of the effectiveness of intervention programs aimed at enhancing these personal resources in professionals exposed to high emotional stress. In these longitudinal intervention designs, an additional post-intervention assessment could be included to examine short-term effects, followed by longer-term follow-ups. Finally, it would be interesting to explore the role of mediating and moderating variables, such as organizational support, work climate, or resilience, which could interact with self-efficacy and coping strategies in predicting psychological symptoms.

## 5. Conclusions

This study provides empirical evidence of the importance of personal resources for the mental health of early childhood care professionals. The findings show that perceived self-efficacy and adaptive coping strategies, particularly cognitive restructuring, positive restructuring and social support, act as protective factors against depression, anxiety and stress. Social withdrawal, however, emerges as a clear risk factor. These results suggest that psychological distress is not solely explained by job demands, but also by how professionals interpret and manage them. From an applied perspective, the data support the need for specific preventive programmes aimed at strengthening professional self-efficacy and training functional emotion-regulation strategies. These programmes should be integrated into the continuing professional development of early intervention teams. Enhancing these resources would promote the psychological well-being of professionals and improve the quality of care provided to children and their families, thereby contributing to the sustainability of the care system.

Finally, this study opens up a relevant area of research within a rarely studied professional group, establishing a foundation for future longitudinal studies and intervention research to confirm causal relationships and evaluate the effectiveness of well-being promotion programmes.

## Figures and Tables

**Table 1:** Sociodemographic data.

Variable	Frequency (n)	Percentage (%)
Sex		
Women	109	87.20
Men	16	12.80
City status		
Single	64	51.20
Married	49	39.20
Common-law couple	9	7.20
Separated/divorced	3	2.40
Profession		
Psychologist	46	36.80
Speech therapist	36	28.80
Physiotherapist	21	16.80
Educator	4	3.20
Social worker	4	3.20
Occupational therapist	4	3.20
Nurse	3	2.40
English teacher	3	2.40
School counsellor	2	1.60
Teacher	2	1.60
Age (Mean ± SD)	33.40 ± 9.70	

**Table 2:** Descriptive statistics for DASS-21, A-EG, and Coping Strategies.

Variable	M	SD
DASS-Depression	3.64	4.31
DASS-Anxiety	3.04	3.83
DASS-Stress	6.73	4.52
Total Depression	13.42	11.46
Total Self-efficacy	13.43	5.71
Positive restructuring	13.73	4.57
Self-criticism	5.26	4.99
Emotional expression	9.80	5.04
Paralysis by despair	11.26	5.48
Social support	12.55	5.25
Cognitive Restructuring	10.52	5.31
Avoidance	5.46	3.58
Social withdrawal	6.47	3.71

M = arithmetic mean; SD = standard deviation.

**Table 3:** Correlations between symptoms of depression, anxiety and stress with coping strategies and overall self-efficacy.

Variables	Depression (DASS-21)	Anxiety (DASS-21)	Stress (DASS-21)
Positive restructuring	−0.46 **	−0.39 **	−0.26 **
Self-criticism	−0.29 **	0.24 **	0.14
Emotional expression	−0.08	−0.10	−0.07
Paralysis by despair	−0.27 **	0.18 *	0.30 **
Social support	−0.43 **	−0.24 **	−0.30 **
Cognitive Restructuring	−0.51 **	−0.40 **	−0.42 **
Avoidance	0.09	0.23 **	0.05
Social withdrawal	0.54 **	0.55 **	0.42 **
Total self-efficacy	−0.59 **	−0.55 **	−0.54 **

Note. Pearson Correlations * *p* < 0.05, ** *p* < 0.01, N = 125.

**Table 4:** Multiple regression for DASS-21, Depression, anxiety and stress variable.

Models and Variables	B	SE	β	*t*	*p*
Model 1. Depression	7.90	1.38		5.73	*p* < 0.001
Positive restructuring	−0.40	0.08	−0.42	−5.06	*p* < 0.001
Self-criticism	−0.04	0.07	−0.05	−0.61	0.54
Emotional expression	0.24	0.07	0.28	3.49	*p* < 0.001
Paralysis by despair	−0.03	0.06	−0.04	−0.54	0.59
Social support	−0.15	0.06	−0.19	−2.39	0.02
Cognitive Restructuring	−0.16	0.08	−0.19	−2.09	0.04
Avoidance	−0.17	0.09	−0.14	−1.84	0.07
Social withdrawal	0.60	0.09	0.52	7.02	*p* < 0.001
Total self-efficacy	−0.36	0.09	−0.45	−3.90	*p* < 0.001
Model 2. Anxiety	6.87	2.02		3.41	*p* < 0.001
Positive restructuring	−0.19	0.08	−0.22	−2.22	0.03
Self-criticism	−0.07	0.06	−0.09	−1.06	0.29
Emotional expression	0.08	0.07	0.11	1.20	0.24
Paralysis by despair	−0.05	0.06	−0.07	−0.86	0.39
Social support	0.07	0.06	0.09	1.05	0.30
Cognitive Restructuring	−0.15	0.08	−0.20	−1.92	0.06
Avoidance	0.06	0.09	0.06	0.67	0.50
Social withdrawal	0.51	0.09	0.50	5.73	*p* < 0.001
Total self-efficacy	−0.14	0.07	−0.20	−1.91	0.06
Model 3. Stress	10.09	2.09		4.82	*p* < 0.001
Positive restructuring	−0.10	0.09	−0.12	−1.14	0.26
Self-criticism	−0.05	0.07	−0.07	−0.74	0.46
Emotional expression	0.04	0.07	0.05	0.54	0.59
Paralysis by despair	0.08	0.06	0.11	1.31	0.19
Social support	−0.07	0.06	−0.09	−1.00	0.32
Cognitive Restructuring	−0.26	0.08	−0.34	−3.27	0.00
Avoidance	−0.03	0.10	−0.03	−0.34	0.73
Social withdrawal	0.42	0.09	0.37	4.54	*p* < 0.001
Total self-efficacy	−0.20	0.07	−0.29	−2.74	0.01

## Data Availability

The datasets generated and analyzed during the current study are not publicly available due to data regulations and for ethical reasons, considering that this information might compromise research participants’ consent because our participants only gave their consent for the use of their data by the original team of investigators. However, collaboration for data analyses can be requested by sending a letter to marobles@ujaen.es, who is the responsible of the investigation.
